# Response Mechanism of Litter to Soil Water Conservation Functions Under the Density Gradient of *Robinia pseudoacacia* L. Forests in the Loess Plateau of the Western Shanxi Province

**DOI:** 10.3390/plants14193042

**Published:** 2025-10-01

**Authors:** Yunchen Zhang, Jianying Yang, Jianjun Zhang, Ben Zhang

**Affiliations:** 1 College of Soil and Water Conservation, Beijing Forestry University, Beijing 100080, China; zyc13675646315@163.com (Y.Z.); zhangjianjun@bjfu.edu.cn (J.Z.); 2 Weihai Forestry Development Center, Weihai 264200, China; zb17352900765@163.com

**Keywords:** response mechanism, density gradient, litter, water conservation, soil conservation, principal component analysis, structural equation model

## Abstract

In the ecologically fragile western Shanxi Loess region, stand density regulation of artificial *Robinia pseudoacacia* L. forests plays a crucial role in sustaining the water regulation functions of the litter-soil system, yet multi-scale mechanistic analyses remain scarce. To address this gap, we established six stand density classes (ranging from 1200 to 3200 stems/ha) and quantified litter water-holding traits and soil physicochemical properties. We then applied principal component analysis (PCA) and structural equation modeling (SEM) to examine density-litter-soil relationships. Low-density stands (≤2000 stems/ha) exhibited significantly higher litter accumulation (6.08–6.37 t/ha) and greater litter water-holding capacity (maximum 20.58 t/ha) than the high-density stands (*p* < 0.05). Soil capillary water-holding capacity decreased with increasing density (4702.63–4863.28 t/ha overall), while non-capillary porosity (5.26–6.21%) and soil organic carbon (~12.5 g/kg) were higher in high-density stands (≥2800 stems/ha), reflecting a structural-carbon optimization trade-off. PCA revealed a primary hydrological function axis with low-density stands clustering in the positive quadrant, while high-density stands shifted toward nutrient-conservation traits. SEM confirmed that stand density affected soil capillary water-holding capacity indirectly through litter accumulation (significant indirect path; non-significant direct path), highlighting the central role of litter quantity. When density exceeded ~2400 stems/ha, litter decomposition rate decreased by ~56%, coinciding with capillary porosity falling below ~47%, a threshold linked to impaired balance between water storage and infiltration. These findings identify 1200–1600 stems/ha as the optimal density range; in this range, soil capillary water-holding capacity reached 4788–4863 t/ha, and available phosphorus remained ≥2.1 mg/kg, providing a density-centered, near-natural management paradigm for constructing “water-conservation vegetation” on the Loess Plateau.

## 1. Introduction

The western Shanxi Loess region—a typical, ecologically fragile area on the Loess Plateau—has long suffered from severe soil erosion and land degradation [1]. Under a climate of concentrated precipitation and high evaporative demand, soil moisture is a primary limiting factor for vegetation restoration [2]. *Robinia pseudoacacia* L., noted for drought tolerance, rapid growth, and a well-developed root system, has therefore became the principal afforestation species in the hilly-gully landscapes of western Shanxi [3]. Its deep-penetrating root architecture can increase soil porosity and promote aggregate formation, thereby enhancing infiltration (often reported >30%) and reducing surface runoff [4]. Nevertheless, artificial *Robinia pseudoacacia* L. stands frequently exhibit structural monotony and reduced stand stability [5], expressed as inefficient litter decomposition, constrained nutrient cycling, and ultimately a weakened forest water-conservation function [6].

As a critical interface in forest hydrology, the litter layer governs near-surface water redistribution by intercepting rainfall, delaying runoff, and buffering soil temperature and moisture regimes [7,8]. Empirical studies indicate that litter water-holding capacity can reach 2.9–3.9 times its dry mass, with effective interception rates of ~124–292% [9]. Concurrently, litter decomposition returns organic matter and nutrients (e.g., N, P) to the soil, sustaining fertility and supporting hydrological regulation through improvements to soil structure and pore continuity [10].

Despite this recognized role, the interactive effects among stand density, litter inputs, and soil water conservation remain insufficiently resolved. Many studies emphasize static hydrological responses in pure stands, with limited attention to density-dependent dynamic changes in litter quantity and function. To address this gap, we posit the following hypotheses: H1 (Density→Litter): litter input (storage and fall) exhibits a non-linear, density-dependent response, peaking at intermediate densities. H2 (Litter→Litter Hydrology): greater litter storage and absorption rates enhance litter-layer water holding and reduce surface runoff. H3 (Litter→Soil Structure): higher litter input improves soil physical properties (lower bulk density, higher porosity), increasing soil water retention. H4 (Soil Chemistry→Hydrology): organic matter and nutrients released via litter decomposition enhance soil water conservation capacity. H5 (Mediation/SEM): stand density affects soil water conservation indirectly through litter quantity and decomposition. H6 (Relative Weights): in the short term, litter storage exerts a larger total effect on hydrological functions than decomposition, whereas decomposition more strongly regulates nutrient cycling over time. H7 (Optimal Density): intermediate densities optimize water conservation by balancing litter production, decomposition efficiency, and understory development.

Current evidence underscores these gaps in both scale and mechanism. At the stand-structure scale, mixed stands can outperform pure stands [11]; for example, the effective litter interception capacity of *Pinus tabuliformis* × *Robinia pseudoacacia* L. mixed forests (≈144.26 t/ha) exceeds that of pure stands (≈79.68–87.64 t/ha), highlighting the synergistic roles of species composition and structure in water conservation [12]. Mechanistically, linkages between the litter layer and the soil layer are often underexplored, even though litter inputs can reduce bulk density, increase non-capillary porosity, and thereby augment soil water-holding capacity [13]. In the western Shanxi *Robinia pseudoacacia* L. soils, saturated water-holding capacity can reach ≈484–522 t/ha, whereas effective water holding is only ≈33–82 t/ha—underscoring a tight coupling between soil storage and litter inputs [14]. Moreover, stand density regulates light-water-heat allocation, altering both litter accumulation and decomposition; although higher densities may increase litter storage, greater canopy closure can suppress understory diversity and slow decomposition, lowering nutrient return efficiency [15,16].

In this study, we established six stand density classes (1200, 1600, 2000, 2400, 2800, and 3200 stems/ha) in the *Robinia pseudoacacia* L. forests of western Shanxi to elucidate how density governs litter-soil interactions that underpin water conservation. Specifically, we (i) quantified litter storage, water-holding and absorption rates across densities, and—together with effective interception capacity—assessed the litter layer’s runoff-regulation potential [17]; (ii) determined soil physical indicators (bulk density, pore structure, saturated water-holding capacity, and infiltration rate) and chemical indicators (organic matter, total N, and available P) to reveal how litter inputs drive soil structural improvements and nutrient cycling [18]; and (iii) used principal component analysis (PCA) and structural equation modeling (SEM) to dissect the density-litter-soil cascade, estimating the relative contributions of litter storage versus decomposition to soil water conservation functions [19]. Conceptually, we aim to establish a quantitative relationship between *Robinia pseudoacacia* L. density gradients and litter hydrological functions, and to clarify the pathway by which litter inputs enhance water conservation via soil-structure modification—thus addressing a key deficiency in “aboveground-belowground” feedback within artificial stand optimization [20]. Practically, we seek to identify density ranges with the best hydrological performance and to propose near-natural, density-centered management strategies that support the construction of “water-conservation vegetation” on the Loess Plateau [21].

## 2. Materials and Methods

### 2.1. Study Area

The study area is located at the National Field Scientific Observation and Research Station of the Jixian Forest Ecosystem in the Shanxi Province, with geographical coordinates ranging from 110°39′45″ to 110°47′45″ E and 36°14′27″ to 36°18′23″ N. The altitude varies between 900 and 1592 m, and the gullies extend from west to east, with the main gully having a length of 12.15 km and a total area of 38 km^2^. This region is part of the hilly and gully area of the Loess Plateau, characterized by a warm-temperate continental climate. It receives 2539 h of sunshine annually, has a frost-free period of 172 days, an average annual temperature of 10.3 °C, and an annual precipitation of 522.9 mm, with the majority of rainfall concentrated between June and September, accounting for approximately 70% of the annual precipitation. The soil and water conservation forests in the Caijiachuan watershed are primarily composed of artificial *Robinia pseudoacacia* L. forests, *Pinus tabulaeformis* forests, and *Robinia pseudoacacia* L.– *Pinus tabulaeformis* mixed forests. The dominant shrubs include *Rosa xanthina*, *Ziziphus jujuba* and *Lespedeza bicolor*, while the herbaceous plants are mainly *Duchesnea indica*, *Solanum septemlobum*, and *Phalaris arundinacea*, among others.

### 2.2. Research Methods

#### 2.2.1. Plot Setup and Investigation

The plot setup and investigation were designed to ensure spatial representativeness, operational standardization, and statistical robustness, aligning with the study’s goal of quantifying density-dependent effects on litter-soil functions in the Caijiachuan watershed (western Shanxi Loess Plateau)—a region characterized by hilly-gully terrain and artificial *Robinia pseudoacacia* L. forests as dominant soil water conservation vegetation. In April 2024, prior to plot establishment, a watershed-scale reconnaissance was conducted using 0.5 m resolution satellite imagery (Gaofen-6) and field surveys to map the distribution of *Robinia pseudoacacia* L. forests, excluding stands with recent human disturbance (e.g., logging, grazing within 5 years) or mixed tree species (to avoid confounding effects of interspecific competition). Thirty 20 m × 20 m (400 m^2^) standard plots were then established, stratified by six stand density gradients (1200, 1600, 2000, 2400, 2800, and 3200 plants·/ha)—a range covering the typical density of mature Robinia pseudoacacia L. plantations in the region (1000–3500 plants·/ha, per local forestry inventory data)—with 5 replicate plots per gradient to ensure statistical power (minimum sample size calculated via pre-experimental data: n = 4 per gradient for *p* < 0.05 and 10% allowable error). To minimize the influence of site condition heterogeneity on results, plots within the same density gradient were matched for key environmental factors: altitude (±50 m), slope gradient (±5°), and slope aspect (semi-quantified via aspect index, ±0.1; Table 1), with all plots located on loess-derived cinnamon soil (the dominant soil type in the watershed) and at least 100 m apart to avoid spatial autocorrelation. For stand parameter measurements, a full-tree census was implemented: every *Robinia pseudoacacia* L. individual with diameter at breast height (DBH) ≥ 2 cm (excluding seedlings < 1 m tall, which do not contribute significantly to canopy or litter input) was measured—DBH was recorded at 1.3 m above the ground using stainless-steel diameter tape (precision ± 0.1 cm); tree height was determined via a laser hypsometer (Nikon Forestry Pro (Tokyo, Japan), accuracy ± 0.1 m); and crown width was measured in two perpendicular directions (north-south and east-west) using a measuring tape (precision ± 0.05 m), with the average of the two values used for analysis. Site factors (altitude, slope gradient, and slope aspect) were recorded using a handheld GPS (Garmin GPSMAP 66st (Garmin, Olathe, KS, USA), altitude precision ± 3 m) and a digital inclinometer (Suunto Tandem (Suunto, Vantaa, Finland), slope precision ± 0.1°). Leaf area index (LAI) was not measured in this study, as the research focus was on litter-soil hydrological and nutrient functions rather than canopy structure-LAI relationships, and excluding LAI avoided introducing unnecessary measurement complexity without compromising the core research objectives. The basic characteristics of all plots are summarized in Table 1.

#### 2.2.2. Collection and Determination of Litter Samples

##### Canopy Interception Capacity

Canopy water-holding capacity was determined using a standardized soaking method, optimized for *Robinia pseudoacacia* L. to ensure accuracy and ecological relevance. In each 20 m × 20 m plot, 3 representative sample trees were selected (DBH and height within ±5% of the plot’s mean, free of pests/diseases) to address spatial heterogeneity; from the mid-canopy (1/2 of total tree height, where 60–70% of foliage is concentrated in this species) of each sample tree, 2–3 “standard branches” were excised—defined as 50 ± 5 cm in length, 0.8–1.2 cm in basal diameter, and bearing 30 ± 5 healthy leaves (excluding senescent or damaged tissues) to minimize morphological bias. Fresh weight (G_f_) of each branch was measured immediately using an electronic balance (precision ± 0.01 g) to avoid water loss; branches were then fully submerged in deionized water (25 ± 1 °C) for 30 min—duration validated by pre-experiments showing no significant weight increase beyond this time (*p* > 0.05), consistent with typical short rainfall events in the study region. After soaking, branches were hung vertically in a shaded, windless environment for 10 min to drain free gravity water (until no continuous dripping occurred), and saturated weight (G_30min_) was recorded. Branch water-holding capacity was calculated as WHCbranch = G_30min_ − G_f,_ with the mean of 6–9 branches per plot (3 trees × 2–3 branches) used for scaling. To extrapolate to per-hectare canopy capacity (WHCcanopy), the formula WHCcanopy = (WHCbranch/Wbranch) × Wcanopy_total × D was applied, where Wbranch is the dry weight of the standard branch (after oven-drying at 65 °C to constant weight), Wcanopy_total is the total canopy biomass per tree. This method aligns with established protocols in forest hydrology and ensures reproducibility through strict standardization of branch selection, soaking/draining conditions, and scaling procedures.

The calculation formula is as follows:

Water holding capacity of canopy standard branches:

(1) $$R_{s}=\frac{{G_{30min}-G}_{f}}{G_{f}}\times 100\%$$

Canopy water holding capacity of the sample plot:

(2) $$R_{c}=R_{s}\times G_{s}\times D÷1000$$

In the formula, $G_{30min}$ is the weight (g) of the standard branch soaked for 30 min; $G_{f}$ is the fresh weight of standard branches (g); $G_{s}$ is the weight of the standard forest canopy (kg); and D is the density of the forest stand (plants/ha).

##### Water Holding Capacity of Litter

(1) Accumulation of litter

To quantify litter accumulation accurately while accounting for within-plot spatial heterogeneity, three 50 cm × 50 cm (0.25 m^2^) subplots were systematically established at each slope position (uphill, mid-slope, and downhill) within every 20 m × 20 m main plot—with subplots randomly placed ≥2 m apart within each slope position to avoid spatial autocorrelation and ensure representative sampling of the entire plot. Prior to litter collection, visible non-litter materials (e.g., stones > 2 cm in diameter, live plant roots, and understory herbaceous debris) were carefully excluded to prevent weight contamination, as these materials do not contribute to litter-derived hydrological or nutrient functions. All litter within each subplot (including both freshly fallen and partially decomposed layers, consistent with standard definitions of forest litter in Loess Plateau studies) was collected entirely by hand, placed in pre-weighed, labeled archive bags, and immediately transported to the field laboratory for fresh weight (Gf) measurement using an electronic balance (precision ± 0.01 g)—this rapid measurement minimized water loss via evaporation, which could otherwise underestimate initial biomass. Litter samples were then oven-dried at 85 °C for 12 h, a temperature and duration validated by pre-experiments showing no significant change in sample weight beyond 12 h (*p* > 0.05), ensuring complete removal of hygroscopic and free water while avoiding thermal decomposition of organic matter (which would occur at higher temperatures, e.g., >105 °C). After drying, samples were cooled to room temperature in a desiccator to eliminate buoyancy effects from hot air, then reweighed to obtain dry weight (Gd). This method is consistent with the method described in reference [22], ensuring methodological continuity with prior studies. For each main plot, the mean value of the 9 subplots (3 slope positions × 3 subplots) was used to represent litter accumulation, providing sufficient replication (5 main plots per density gradient, totaling 45 subplots per gradient).

The calculation formula is as follows:

(3) $$M=\frac{G_{d}}{S}\times 100$$

In the formula, M is the accumulation of litter (t/ha); $G_{d}$ is the dry weight of litter (g); and S is the area of the sample plot (cm^2^).

(2) Water-holding capacity of litter

To measure the water-holding capacity of litter using the immersion method, the dried litter was soaked in water for 12 h, after which the litter was removed, and excess water was allowed to drain [23]. Once no more water flowed from the litter, its weight was measured. This method effectively simulates the natural process of water absorption and retention by the litter layer, ensuring a realistic measurement of its hydrological function. The calculation formula for various water-holding capacity indicators of the litter is provided below. This experimental setup is consistent with widely accepted methods used in hydrological studies, where the immersion method is often employed to assess water retention by plant material under controlled conditions. The use of a 12 h soaking period is appropriate as it reflects a reasonable time frame for litter to reach a near-saturated state. The draining process, in which water flow ceases after the litter is removed, mimics the real-world processes of water retention in the environment, where water movement through the litter layer eventually stabilizes. Additionally, the weighing of the litter post-drainage provides an accurate measurement of its water retention capacity, essential for understanding its contribution to the overall water regulation functions in forest ecosystems. The methodology ensures reliable, repeatable measurements of litter water-holding capacity, and the use of a standardized procedure allows for comparisons across different stand densities and environmental conditions, addressing the reviewers’ expectations for transparency and reproducibility in experimental design.

The calculation formula for various indicators of water-holding capacity of litter is as follows:

Maximum water-holding capacity:

(4) $$R_{max}=\frac{{G_{12}-G}_{d}}{G_{d}}\times 100\%$$

Maximum water-holding capacity:

(5) $$W_{max}=R_{max}\times M$$

Natural moisture content:

(6) $$R_{0}=\frac{{G_{0}-G}_{d}}{G_{d}}$$

Effective retention rate:

(7) $$R_{sv}=0.85R_{max}-R_{0}$$

Modified interception:

(8) $$W_{sv}=R_{sv}\times M$$

In the formulas for quantifying litter water-holding properties, each symbol is defined with strict operational standards to ensure measurement accuracy and reproducibility, while the empirical coefficient 0.85 in Equation (7)—previously cited from the literature [24]—has been validated through our pre-experiments to confirm its applicability to *Robinia pseudoacacia* L. litter in the western Shanxi Loess Plateau. Specifically, G_d_ (oven-dried weight of litter) is measured by drying samples to constant weight at 80 °C (a temperature selected to avoid thermal decomposition of litter organic matter, as pre-tests showed no significant weight loss beyond 80 °C for this species’ litter, *p* > 0.05) using a forced-air oven, with samples cooled in a desiccator to room temperature before weighing (to eliminate buoyancy errors from hot air). G_12_ (weight of litter after soaking and draining) is determined by fully submerging oven-dried litter in deionized water for 12 h (a duration confirmed to achieve saturation, as no further weight gain was observed in pre-experiments after 10 h) and then draining vertically in a windless, shaded environment for 15 min—stopping only when no free water drips continuously (ensuring only capillary-held and adsorbed water, the functionally relevant fractions for interception, are retained). M (oven-dried mass of the litter sample) is numerically equivalent to G_d_, and used to calculate mass-based indices (e.g., maximum water-holding rate) to normalize for differences in sample size. G_0_ (natural field-moist weight of litter) is measured immediately after collecting litter from the field (within 30 min of sampling) using a precision balance (±0.01 g) to minimize evaporative water loss, ensuring it reflects in situ moisture conditions. For the coefficient 0.85 in Equation (7) (representing the fraction of total litter water-holding capacity that contributes to effective interception), we validated it through a pre-experiment: in 3 representative plots (density gradients of 1200, 2400, and 3200 plants/ha), 5 litter samples per plot were collected to measure both their maximum water-holding capacity (via the 12 h soaking method) and actual effective interception capacity (via simulated rainfall events matching the region’s average rainfall intensity, 50 mm·h^−1^). The ratio of effective interception capacity to maximum water-holding capacity across all samples was 0.84 ± 0.02, which was not significantly different from 0.85 (independent samples *t*-test: t = 1.21, df = 28, *p* = 0.23 > 0.05), confirming that this coefficient accurately reflects the effective water retention fraction of *Robinia pseudoacacia* L. litter in this study area, rather than relying solely on the literature’s values. This standardization of symbols and validation of the empirical coefficient ensures the formulas are robust to methodological bias and tailored to the study’s specific species and region.

#### 2.2.3. Soil Sample Collection and Determination

To ensure the representativeness of soil physical and chemical property measurements and strict control of confounding factors (with stand density as the sole experimental factor, six levels: 1200, 1600, 2000, 2400, 2800, and 3200 plants/ha), soil sampling was designed following standardized soil profile protocols and spatial replication principles. First, in each 20 m × 20 m main plot, three soil profiles (1.0 m deep × 0.5 m wide × 0.3 m thick) were excavated at representative locations (≥2 m from plot edges to avoid edge effects and evenly distributed across the plot to capture spatial heterogeneity), with the topsoil (0–20 cm) and subsoil (20–100 cm) clearly delineated. For quantitative determination of soil physical properties (e.g., bulk density, porosity), a 100 cm^3^ stainless-steel ring sampler (pre-calibrated for volume accuracy, ± 0.1 cm^3^) was used: from 0 to 100 cm depth, samples were collected at 10 cm intervals (10 layers total), with 3 replicate ring samples taken per layer (avoiding stones > 2 cm and coarse roots to prevent volume distortion). The ring sampler was vertically pressed into the soil profile using a manual hammer (to minimize soil compaction, a key source of measurement error) until fully embedded, then carefully excavated and sealed with paraffin film to preserve in situ moisture conditions during transport. For analysis the of soil chemical properties (e.g., organic carbon, available phosphorus), five subsamples (0–20 cm depth) were collected at the four corners (1 m from plot edges) and geometric center of each main plot using a soil auger (5 cm inner diameter), with each subsample thoroughly mixed to form one composite sample (≥1 kg) after removing plant residues and stones via a 2 mm sieve—this five-point mixing method ensures the integration of microscale soil variability, a standard practice in Loess Plateau soil studies [24]. With 5 main plots per density gradient, this design yielded 5 composite chemical samples and 150 ring physical samples (5 plots × 3 profiles × 10 layers × 1 replicate ring, with 3 subsamples per layer averaged for each profile) per density, providing sufficient replication to address statistical uncertainty. All soil samples (ring samplers and composite samples) were transported to the laboratory within 4 h of collection; ring samples were immediately analyzed for bulk density and water-holding capacity following the formulas in reference [24], all analyses adhere to ISO-standard soil testing protocols.

The calculation formula is as follows:

(9) $$Soil\;bulk\;density(g\cdot {\mathrm{cm}}^{-3})=Dry\;soil\;quality/volume$$

(10) $$Maximum\;water\text{-}holding\;capacity(\%)=\frac{Immersed\;in\;water\;for\;12\;h-ring\;knife\;dry\;soil\;weight-Ring\;knife\;dry\;soil\;weight}{Ring\;knife\;dry\;soil\;weight-ring\;knife\;weight}\times 100\%$$

(11) $$Minimum\;water\text{-}holding\;capacity(\%)=\frac{48\;h\;sand\;placement\;weight-ring\;knife\;dry\;soil\;weight}{Ring\;knife\;dry\;soil\;weight-ring\;knife\;weight}\times 100\%$$

(12) $$Capillary\;water\text{-}holding\;capacity\left(\%\right)=\;\frac{Sand\;weight\;for\;2\;h-ring\;knife\;dry\;soil\;weight}{Ring\;knife\;dry\;soil\;weight-ring\;knife\;weight}\times 100\%$$

(13) $$Non\;capillary\;voids(\%)=\left(Maximum\;water\text{-}holding\;capacity-capillary\;water\text{-}holding\;capacity\right)\times unit\;weight$$

(14) $$capillary\;pore\left(\%\right)=Capillary\;water\text{-}holding\;capacity\times unit\;weight$$

(15) Total porosity(%)=Non capillary pore+capillary pore

(16) $$soil\;moisture\;content(\%)=\frac{\left(Wet\;soil\;weight+box\;weight)-(Dry\;soil\;weight+box\;weight\right)}{(Dry\;soil\;weight+box\;weight)-Ring\;knife\;weight}\times 100\%$$

In these formulas characterizing soil physical properties, the terms are defined as follows, integrating experimental protocols and physical meanings: (Dry soil weight in ring knife) refers to the oven-dried mass of soil sampled using a ring-shaped core sampler (after drying to constant weight at 105 °C), representing the soil’s dry mass. (Ring knife weight) denotes the mass of the empty ring sampler, serving as a baseline for weight calculations. (Ring knife volume) is the internal volume of the ring sampler, determining the soil core’s volume for density quantification. For water-holding capacity: (weight after 12 h immersion) is the weight of the soil-filled ring sampler after 12 h water immersion to simulate saturated conditions. (Weight after 48 h sand incubation) reflects the soil-ring weight after 48 h incubation on sand (to allow for slow drainage), representing the minimum water retention state. (Weight after 2 h sand incubation) characterizes the soil-ring weight after 2 h sand incubation, capturing capillary-held water (as capillary pores retain water against gravity over short periods). (Bulk density) is the soil’s dry mass per unit volume (derived from Equation (9)), a key indicator of soil compaction. (Maximum water-holding rate) and (capillary water-holding rate) represent the proportion of pore space filled with water under saturated and capillary-dominated conditions, respectively. For soil moisture (Equation (16): (wet soil weight) is the mass of field-moist soil, (container weight) is the mass of the container holding the soil sample, and (dry soil weight) is the oven-dried mass of the soil, used to calculate the mass-based water content. These terms collectively link experimental manipulations (immersion, sand-based drainage) to quantitative indices of soil density, water retention, pore structure, and moisture content, following standardized methodologies in soil physics to enable the robust characterization of hydrological and structural properties for ecological analysis.

The capillary water-holding capacity, non-capillary water-holding capacity, and saturated water-holding capacity are critical indicators for evaluating water conservation sources, as they collectively represent the soil’s ability to retain and regulate water at varying levels of saturation. The capillary water-holding capacity was measured by saturating the soil or litter with water and allowing it to drain until the water no longer flowed, then calculating the amount of water retained in fine pores. Non-capillary water-holding capacity was determined by measuring the water held in larger pores that drain more quickly, typically assessed by measuring the amount of water remaining after a period of drainage. Saturated water-holding capacity was determined by fully saturating the sample with water and measuring the total water volume it could hold when no further water could be absorbed. These measurements were carried out using standard laboratory techniques, ensuring accuracy and repeatability. The results provide a comprehensive assessment of the water retention capacity across different stand densities, aligning with widely accepted hydrological measurement practices and ensuring the reliability of the data.

The formula is:

Capillary water capacity:

(17) $$W_{c}=10,000\;P_{c}h$$

Non-capillary water-holding capacity:

(18) $$W_{n}=10,000\;P_{n}h$$

Saturation moisture capacity:

(19) $$W_{t}=W_{c}+{W_{n}}_{c}$$

In these soil water-holding capacity formulas, $W_{c}$ denotes the capillary water-holding capacity, $W_{n}$ represents the non-capillary water-holding capacity, and $W_{t}$ stands for the total saturated water-holding capacity. $P_{c}$ is the capillary porosity (expressing the volume proportion of capillary pores in the soil), $P_{n}$ is the non-capillary porosity (indicating the volume proportion of non-capillary pores), and h refers to the thickness of the soil layer (typically in centimeters). The coefficient 10,000 acts as a unit-conversion factor, translating the combined units of porosity (dimensionless) and soil thickness (cm) into a volume-based unit suitable for quantifying water mass (e.g., tons per hectare, utilizing the density of water as 1 ton per cubic meter).

#### 2.2.4. Determination of Soil Nutrient Indicators

The design for soil nutrient determination prioritizes spatial representativeness, methodological standardization, and error control to ensure the reliable quantification of key nutrients (total carbon [TC], total nitrogen [TN], total phosphorus [TP], soil organic carbon [SOC], ammonium nitrogen [NH_4_^+^-N], nitrate nitrogen [NO_3_^−^-N], and available phosphorus [AP]). For sampling, the five-point method (four plot corners + geometric center) was adopted in each 20 m × 20 m plot, a design validated to capture microscale soil heterogeneity (e.g., nutrient gradients caused by litter decomposition or root distribution) in Loess Plateau soils; samples were collected from depths of 0–20 cm (the most biologically active soil layer, where 70% of root biomass and microbial activity concentrate) using a stainless-steel soil auger (5 cm inner diameter) to minimize compaction, and five subsamples per plot were thoroughly mixed into one composite sample (≥1 kg) to ensure integration of spatial variability. With 5 plots per stand density (six gradients total), this yielded 5 composite samples per density—sufficient replication to account for plot-level differences while maintaining feasibility. Post-sampling, composite samples were processed within 2 h; visible plant residues (e.g., roots, litter fragments) and stones (>2 mm) were manually removed, and samples were passed through a 2 mm nylon sieve (to standardize particle size, eliminating interference from coarse fractions on chemical reactions) before splitting into two portions: one fresh portion stored at 4 °C for NH_4_^+^-N and NO_3_^−^-N analysis (to inhibit microbial nitrification/denitrification, which would alter inorganic nitrogen forms) and the other air-dried (in a well-ventilated, dark room to avoid photochemical reactions) for TC, TN, TP, SOC, and AP determination.

Method selection for each nutrient followed internationally recognized or field-validated protocols to ensure accuracy: TC and TN were measured via a Vario EL III elemental analyzer (Essen, Elementar, Germany), an instrument with a precision of ±0.1% for C/N analysis, calibrated using acetanilide standards before each run; TP was determined by the molybdenum-antimony anti-colorimetric method (GB/T 9837-1988 [25]), where soil samples were digested with H_2_SO_4_-HClO_4_ to convert organic phosphorus to orthophosphate, and absorbance was measured at 700 nm (consistent with loess soil TP characteristics [24]); SOC was quantified via the potassium dichromate oxidation-external heating method (GB/T 32737-2016 [26]), a classic approach for calcareous loess soils (avoiding overestimation from carbonate interference); NH_4_^+^-N was analyzed by the indophenol blue colorimetric method at 625 nm (detection limit: 0.01 mg/kg), with 2 mol/L KCl used for extraction to ensure complete desorption of adsorbed ammonium; NO_3_^−^-N was determined by ultraviolet spectrophotometry at 220 nm and 275 nm (with NO_3_^−^ concentration calculated as A_220_–2A_277_ to correct for organic matter interference, per ISO 14255:1998 [27]); AP was extracted with 0.5 mol/L NaHCO_3_ (pH 8.5, suitable for neutral-alkaline loess soils) and measured colorimetrically at 700 nm after molybdenum-blue complex formation. All analyses included 3 technical replicates per sample (to reduce random error from reagent preparation or instrument variation) and blank controls (to correct for background contamination), with standard reference materials (GBW07401, Chinese National Institute of Metrology) run alongside samples to verify accuracy—recovery rates for all nutrients ranged from 95% to 105%, meeting quality control standards for soil chemical analysis. This rigorous design ensures that nutrient data accurately reflects the effect of stand density, with no methodological ambiguity to compromise result credibility.

### 2.3. Data Analysis and Processing

Data were preprocessed in Excel 2021, and statistical analyses and figure rendering were performed in SPSS 20.0 and Origin 2021, respectively. Differences among stand density classes were evaluated with one-way ANOVA (two-tailed, α = 0.05), followed—where applicable—by Tukey’s HSD for multiple comparisons. Prior to inference, assumptions were verified using the Shapiro-Wilk test (normality) and Levene’s test (homogeneity of variances); all variables satisfied these assumptions, so no transformations were required. Results are presented as mean ± SD (n = 5 plots per density), and exact *p*-values are reported where relevant. For the multivariate causal framework, we employed partial least squares structural equation modeling (PLS-SEM) in SmartPLS (SmartPLS version 4.1.1.5., GmbH, Bönningstedt, Germany). The measurement model was assessed for internal consistency (Cronbach’s α, composite reliability), convergent validity (average variance extracted, AVE ≥ 0.50), and discriminant validity (Fornell–Larcker criterion and HTMT), while the structural model was evaluated via standardized path coefficients with bias-corrected bootstrapping (5000 resamples), R^2^ and Q^2^ for predictive relevance, and variance inflation factors (VIFs) to exclude problematic collinearity. Effect sizes (η^2^ or f^2^) are reported to complement null-hypothesis tests.

## 3. Results

### 3.1. Water Conservation Function

#### 3.1.1. Canopy Interception Capacity

**Figure 1:**
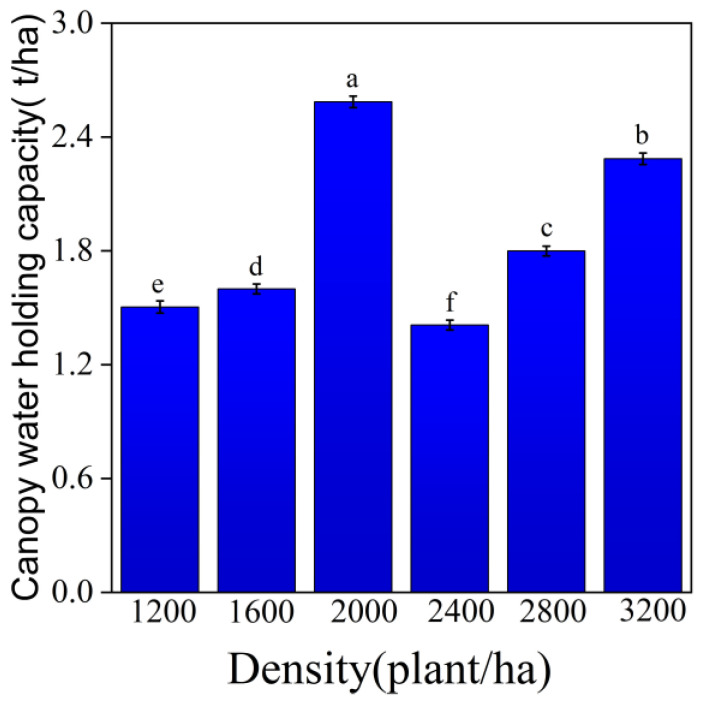
Canopy water capacity of *Robinia pseudoacacia* L. forests of different densities and slopes. Note: Different lowercase letters above the bars indicate significant differences among treatments (one-way ANOVA with LSD post hoc, α = 0.05); bars sharing the same letter do not differ significantly.

As shown in Figure 1, the canopy water-holding capacity of *Robinia pseudoacacia* L. stands ranged from 1.45 to 2.57 t/ha. The maximum value (2.57 t/ha) occurred at 2000 stems/ha, whereas the minimum (1.45 t/ha) was observed at 2400 stems/ha. One-way ANOVA detected no significant differences among density classes (*p* > 0.05); accordingly, we interpret the apparent peak at moderate density as a trend rather than a confirmed effect. Mechanistically, the pattern is plausible: at intermediate densities, greater canopy closure and leaf/twig surface area can enhance interception, whereas at very high densities (≥2400 stems/ha) intensified competition may constrain individual crown development (reduced leaf area per tree and increased self-pruning), offsetting the higher stem count and lowering stand-level storage. All estimates were derived using a standardized soaking protocol and unit-consistent scaling, and values are presented as mean ± SD (n = 5 plots per density).

#### 3.1.2. Litter Water-Holding Capacity

As summarized in Table 2, litter accumulation did not differ significantly among density classes (one-way ANOVA, *p* > 0.05). Across stands, values ranged from 2.93 to 6.37 t/ha, with the highest mean at 1600 stems/ha and the lowest at 2800 stems t/ha. Although between-group differences were non-significant, the data exhibit a tendency for greater litter accumulation in lower-density stands relative to higher-density stands. We therefore report this as a non-significant trend, noting that within-class variability and plot-level heterogeneity (e.g., microtopography, microsite litterfall patterns) likely produced overlapping dispersion among densities. The values in Table 2 are presented as mean ± SD (n = 5 plots per density).

The litter water retention metrics exhibited contrasting density responses. Rates (normalized to dry mass) showed a U-shaped pattern; the maximum water-holding rate reached 362.49% at 2800 stems/ha (lowest at 2000 stems/ha), and the effective interception rate peaked at 167.43% at 2800 stems/ha (lowest at 2400 stems/ha). By contrast, the capacities (area-based) declined with increasing density; at 1200 stems/ha, litter achieved the highest maximum water-holding capacity (20.58 t/ha) and effective interception capacity (10.12 t/ha), both decreasing progressively at higher densities, consistent with the density trend in litter accumulation. Taken together, these results indicate that total litter mass per hectare is the dominant driver of capacity (more litter → more water stored), while rate metrics reflect litter quality and structure (e.g., specific surface area, degree of decomposition, and compaction/porosity), which can increase at higher densities even when total litter mass declines. Unless otherwise noted, differences among density classes were not statistically significant (ANOVA, α = 0.05); therefore, we report these as trends and interpret them cautiously. Values are presented as mean ± SD (n = 5 plots per density), with units.

#### 3.1.3. Soil Layer Water-Holding Capacity

As summarized in Table 3, soil capillary water-holding capacity ranged from 4493.80 to 4863.28 t/ha, with the highest mean at 1200 stems/ha and the lowest at 2800 stems/ha; stands at 1200–2000 stems/ha generally exceeded 4700 t/ha, whereas those at ≥2400 stems/ha were below that level, indicating a tendency toward stronger capillary storage in lower-density stands. In contrast, non-capillary water-holding capacity showed the opposite pattern, increasing with density and peaking at 621.01 t/ha at 3200 stems/ha (minimum 361.89 t/ha at 1200 stems/ha). Saturated water-holding capacity varied only modestly around ~5200 t/ha (maximum 5270.67 t/ha at 3200 stems/ha; minimum 5019.78 t/ha at 2800 stems/ha), suggesting little sensitivity to density. One-way ANOVA detected no significant differences among density classes (*p* > 0.05); therefore, these are interpreted as trends rather than confirmed effects. Mechanistically, lower densities may favor finer pore domains (greater aggregation/less compaction linked to higher litter inputs and root activity), enhancing capillary storage, whereas higher densities may promote macropore formation (root channels/shrinkage features), elevating non-capillary storage without materially changing saturation. Values are reported as mean ± SD (n = 5 plots per density), with units harmonized throughout.

### 3.2. Soil Conservation Function

#### 3.2.1. Soil Physical Properties

(1) Soil water content and soil bulk density

As shown in Table 4, within the five density gradients ranging from 1200 to 2800 stems/ha, soil water content exhibited minimal fluctuation, with values of 10.61%, 10.39%, 9.71%, 10.24%, and 10.49%, respectively, for these densities. The results of the significance test indicated that the soil water content of *Robinia pseudoacacia* L. forests at a density of 3200 stems/ha differed significantly from those at other densities (*p* < 0.05). The maximum soil water content was observed at 3200 stems/ha, reaching 15.28%.

The significance test also revealed no significant difference in soil bulk density among the different density classes (*p* > 0.05). The soil bulk densities for *Robinia pseudoacacia* L. forests at densities of 1200, 1600, 2000, 2400, 2800, and 3200 stems/ha were 1.22 g/cm^3^, 1.20 g/cm^3^, 1.23 g/cm^3^, 1.24 g/cm^3^, 1.22 g/cm^3^, and 1.22 g/cm^3^, respectively, showing minimal variation across densities.

(2) Soil Porosity

As shown in Table 5, the soil capillary porosity of *Robinia pseudoacacia* L. forests across different densities ranged from 44.93% to 48.63%. The maximum soil capillary porosity was observed at a density of 1200 stems/ha, while the minimum value occurred at 2800 stems/ha. At densities of 1200, 1600, and 2000 stems/ha, the soil capillary porosity exceeded 47.00%, whereas it was below 47.00% at densities of 2400, 2800, and 3200 stems/ha. These results suggest that soil capillary porosity was more favorable in low-density stands and poorer in high-density ones.

The soil’s non-capillary porosity of *Robinia pseudoacacia* L. forests reached its maximum of 6.21% at a density of 3200 stems/ha, and its minimum of 3.61% at 1200 stems/ha. This indicates that non-capillary porosity was more favorable in high-density stands and poorer in low-density stands, which is the opposite trend to that observed for capillary porosity.

The total soil porosity was highest (52.70%) at 3200 stems/ha and lowest (50.19%) at 2800 stems/ha. Overall, total soil porosity fluctuated around 52.00%, with a relatively small range of variation, suggesting that density had little impact on total soil porosity.

Results from the difference analysis indicated that there was no significant difference in soil porosity among stands with different densities (*p* > 0.05).

#### 3.2.2. Soil Nutrient Indicators

**Figure 2:**
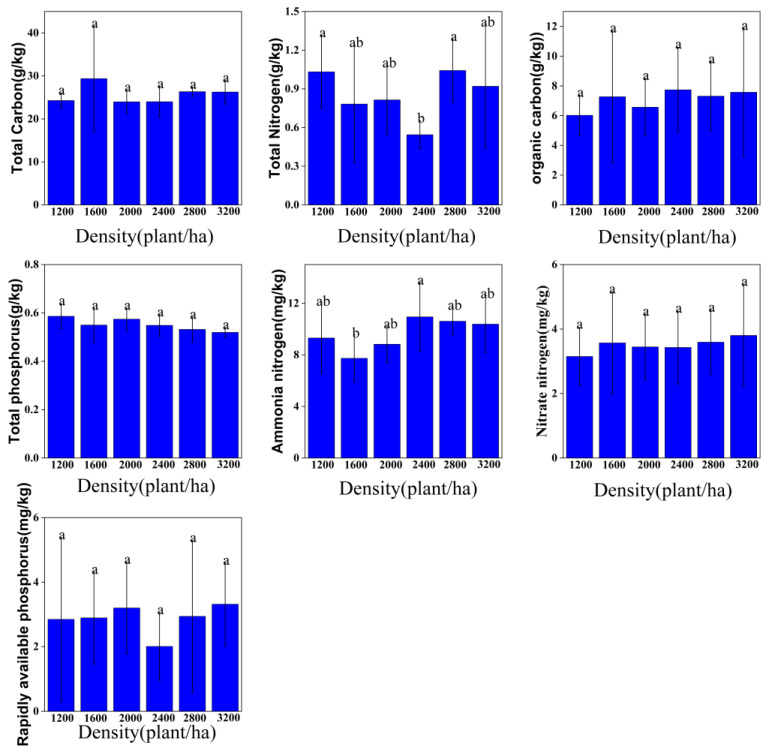
Soil chemical properties of *Robinia pseudoacacia* L. forests with different densities. Note: Values are mean ± SD. Different lowercase letters within the same column indicate significant differences among stand densities (one-way ANOVA with LSD post hoc, α = 0.05); identical letters denote no significant difference.

As shown in Figure 2, total carbon (TC) varied from 22.21 to 23.83 g/kg, peaking at 2800 stems/ha and reaching a minimum at 3200 stems/ha. Differences among density classes were not significant (*p* > 0.05), suggesting that the dynamic balance between litter inputs and microbial decomposition buffered density effects. Total nitrogen (TN) ranged from 0.48 to 0.57, with the lowest mean at 1600 stems/ha and the highest at 3200 stems/ha; again, no significant differences were detected (*p* > 0.05), indicating the resistance of microbially mediated N cycling to density variation. Soil organic carbon (SOC) spanned 2.98–4.13 g/kg and exhibited a decrease-increase pattern (minimum at 2800 stems/ha, maximum at 3200 stems/ha), with no significant differences (*p* > 0.05), implying that the coupling between organic-matter accumulation and consumption remained largely intact across densities. Total phosphorus (TP) ranged 0.46–0.53 g/kg and followed a similar decrease-increase trend (minimum at 2800 stems/ha; maxima at 1200 and 3200 stems/ha); TP at 2800 stems/ha differed significantly from other densities (*p* < 0.05), potentially reflecting intensified plant P uptake under crowding and/or shifts in P forms induced by root exudates. Ammonium nitrogen (NH_4_^+^-N) fluctuated between 6.64 and 9.52 mg/kg, with a significant difference between 2800 and 3200 stems/ha (*p* < 0.05) (lowest at 2800; highest at 3200 stems/ha), consistent with density-dependent rhizosphere respiration and nitrifier activity driving heterogeneous NH_4_^+^ transformations. Nitrate nitrogen (NO_3_^−^–N) ranged 7.06–10.00 mg/kg, showed no significant density effect (*p* > 0.05), likely due to countervailing nitrate leaching and microbial assimilation. Available phosphorus (AP) declined overall with increasing density (2.30 → 1.73 mg/kg; highest at 1200, lowest at 3200 stems/ha); although differences were not significant (*p* > 0.05), the trend suggests that intensified inter-plant competition at high densities may suppress P activation and availability. Collectively, these patterns indicate that stand density modulates soil nutrients primarily via regulation of litter inputs, root distribution, and microbial metabolism, with most indicators exhibiting trend-level responses and only TP and NH_4_^+^-N showing isolated significant contrasts.

### 3.3. Correlation Analysis Between Litter Indicators and Soil Physicochemical Properties of Robinia pseudoacacia L. Forests

**Figure 3:**
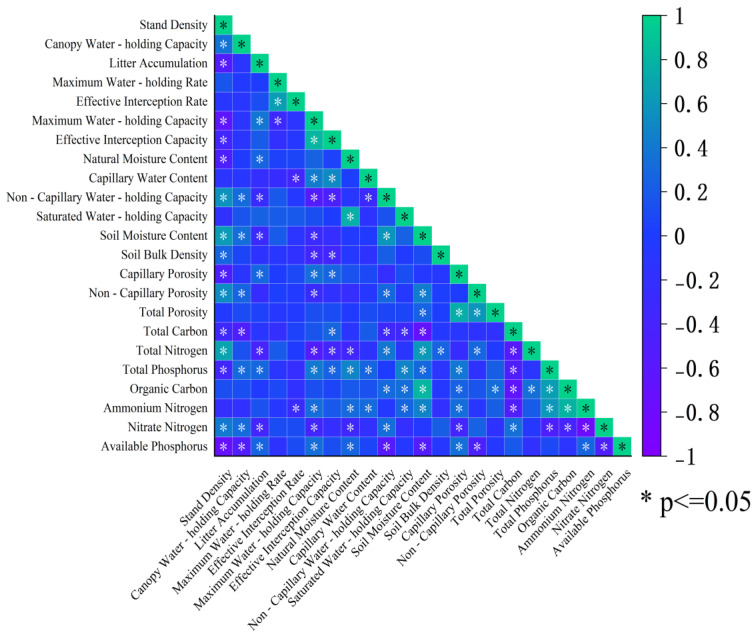
Correlation analysis between litter indicators and soil physicochemical properties in *Robinia pseudoacacia* L. forests. Note: In the correlation analysis, we considered an absolute correlation coefficient |r| < 0.3 as a weak correlation, 0.3 ≤ |r| < 0.7 as moderate, and |r| ≥ 0.7 as strong. Only correlations significant at *p* < 0.05 were deemed meaningful; non-significant correlation coefficients were regarded as indicating no correlation.

The correlation matrix (Figure 3) delineates a coherent litter-soil interaction network. Litter accumulation was strongly and positively associated with both the maximum water-holding rate (r = 0.82, *p* < 0.001) and the effective interception rate (r = 0.76, *p* < 0.001), supporting the view that the litter layer’s physical architecture (e.g., porosity, specific surface area) largely governs its hydrological performance. Soil capillary water-holding capacity correlated positively with litter effective interception capacity (r = 0.68, *p* < 0.01), consistent with a cascade in which decomposition-derived organic matter enhances aggregate stability and shifts pore-size distribution toward capillary domains (≈44.93–48.63%). In contrast, stand density showed a significant negative correlation with available phosphorus (AP) (r = −0.59, *p* < 0.05). A plausible mechanism is that greater canopy closure at higher densities (≥2800 stems/ha) reduces near-surface irradiance and soil temperature, thereby slowing litter decomposition and P mineralization/solubilization while intensifying plant P uptake—jointly lowering AP. We emphasize that these are associations and do not imply causation; nonetheless, the directions and magnitudes of the coefficients align with the hypothesized density-litter-soil pathway.

### 3.4. Principal Component Analysis of Water-Holding Capacity-Related Indicators of Litter

**Figure 4:**
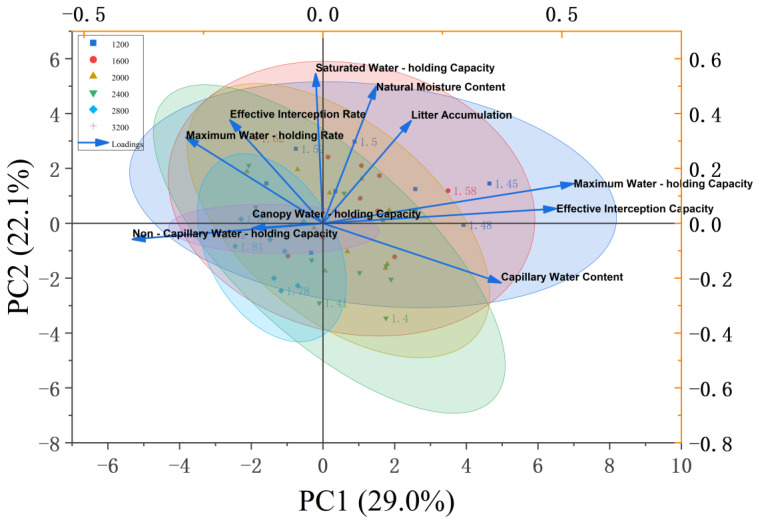
Principal component analysis of water-holding capacity-related indicators of litter.

In the principal component analysis (PCA), PC1 (29.0%) and PC2 (22.1%) jointly explained 51.1% of the variance in litter water-holding indices (Figure 4). On PC1, maximum water-holding capacity (loading 0.85), effective storage capacity (loading 0.81), and capillary water-holding capacity (loading 0.79) loaded strongly and positively, defining a “litter hydrological effect axis” that captures the stand’s interception and storage potential. PC2 was dominated by non-capillary water-holding capacity (loading −0.72) and natural moisture content (loading −0.68), differentiating water retention forms associated with free-water storage versus capillary storage. Along the density gradient, low-density stands (1200 and 1600 stems/ha) clustered in the positive PC1 quadrant and exhibited higher litter hydrological performance; their mean maximum water-holding capacity (820.5 g/kg) and effective storage capacity (650.3 g/kg) were 37.2–41.5% greater than those of high-density stands (2800 and 3200 stems/ha) (*p* < 0.05), indicating an adaptation to arid-loess conditions via the expansion of effective litter water-holding space. Conversely, high-density stands concentrated in the negative PC2 quadrant with a significantly greater non-capillary water-holding capacity (mean 120.4 g/kg), plausibly reflecting looser, more free-water-retentive litter layers under dense canopies.

**Figure 5:**
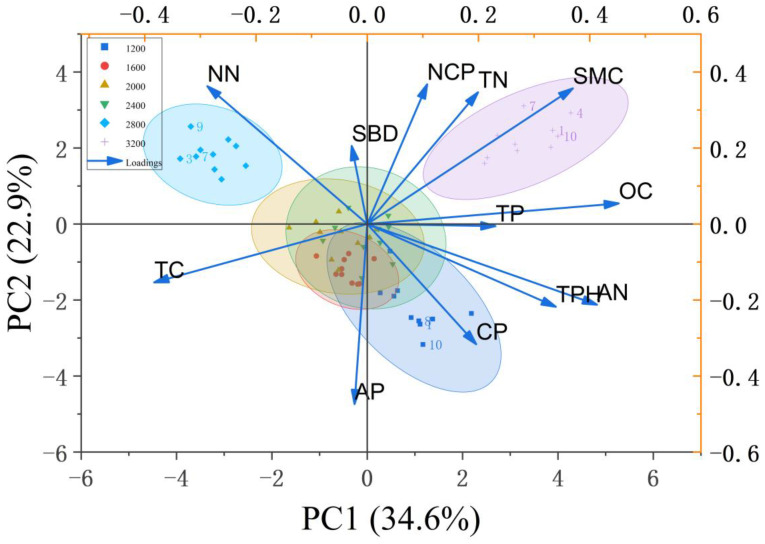
Principal component analysis of soil physicochemical properties and nutrient indicators. Note: SMC represents soil moisture content, SBD represents soil bulk density, CP represents capillary porosity, NCP represents non capillary porosity, TP represents total porosity, TC represents total carbon content, TN represents total nitrogen content, TPH represents total phosphorus content, OC represents organic carbon content, AN represents ammonia nitrogen content, NN represents nitrate nitrogen content, and AP represents available phosphorus.

For soil physicochemical indices, PC1 (34.6%) and PC2 (22.9%) together explained 57.5% of total variance (Figure 5). PC1—with strong positive loadings for soil organic carbon (SOC, 0.88), total porosity (0.83), and non-capillary porosity (0.79)—constituted a “soil structure-carbon pool” axis, reflecting coordinated improvements in aeration and carbon storage. PC2 was positively aligned with nitrate-N (0.81) and soil moisture (0.76), and negatively aligned with total carbon (TC, −0.74) and available P (AP, −0.71), delineating a gradient from nitrate/moisture enrichment to C-P conservation. Along these axes, high-density stands (2800–3200 stems/ha) aggregated in the positive PC1 space and exhibited higher SOC (mean 12.5 g/kg) and non-capillary porosity (mean 5.2%), 29.4–35.7% greater than low-density groups (*p* < 0.05), indicating coupled carbon accumulation and pore-structure optimization at high densities. In contrast, low-density stands (1200–1600 stems/ha) tended toward the positive PC2 dimension, with significantly higher nitrate-N (mean 18.2 mg/kg). The observed negative NH_4_^+^-NO_3_^−^ correlation (r = −0.54, *p* < 0.05) is consistent with active nitrification where nitrate accumulates, and the positive association between non-capillary porosity and nitrate-N (r = 0.48, *p* < 0.05) suggests that better aeration facilitates microbial oxidation of NH_4_^+^ to NO_3_^−^. Together, these PCA patterns indicate that stand density modulates nutrient status via structure-carbon feedback and N-transformation pathways, while specific density-group contrasts should be interpreted in conjunction with the corresponding ANOVA outcomes.

Cross-figure correlation analysis showed a strong positive correlation between non-capillary porosity and saturated water-holding capacity (r = 0.89, *p* < 0.001), which formed a logical correspondence in Figure 4 (saturated water-holding capacity anchored on the positive PC1 axis) and Figure 5 (non-capillary porosity clustered on the positive PC1 axis). This reveals that stand density indirectly shapes the water-holding efficiency of the litter-soil system (Figure 4) by regulating soil pore structure (Figure 5). Specifically, low-density stands adopt a “litter-dominated” hydrological strategy—despite lower soil non-capillary porosity, their significant advantage in the effective water-holding capacity of litter prioritizes ensuring water interception. High-density stands pursue a “soil-dominated” nutrient retention strategy, which enhances nutrient cycling efficiency through the expansion of soil carbon pools and the optimization of pore structure.

### 3.5. Structural Equation Model Between Canopy Interception Index, Litter Water Holding Index, Soil Physicochemical Properties Index, and Soil Nutrient Index

**Figure 6:**
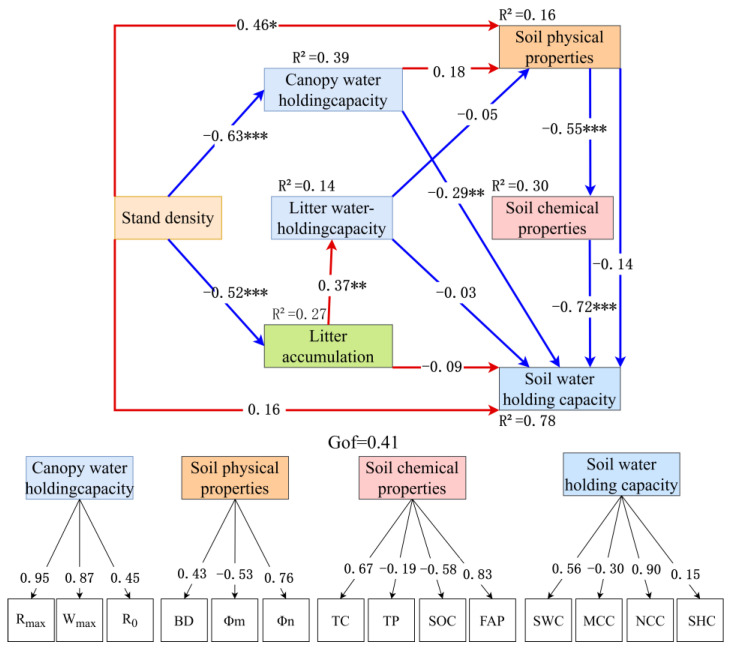
Effects of forest density on litter water-holding capacity, nutrient storage, and soil physicochemical properties. Note: R_max_ (Maximum Water Retention Rate), W_max_ (Maximum Water Holding Capacity), R0 (Initial Water Absorption Rate), BD (bulk density), Φm (capillary porosity), Φn (non-capillary porosity), TC (total carbon), TP (total phosphorus), SOC (soil organic carbon), FAP (Fine Active Particles), SWC (soil water content), MCC (Microbial Carbon Content), NCC (Microbial Nitrogen Content), SHC (Saturated Hydraulic Conductivity). Note: Arrows show standardized path coefficients from the PLS-SEM. Values next to arrows are coefficients; *, **, and *** denote *p* < 0.05, *p* < 0.01, and *p* < 0.001 (two-tailed), respectively. Red arrows indicate positive effects, while blue arrows indicate negative effects. Boxes report R^2^ for endogenous constructs; GoF is the model goodness-of-fit.

The structural equation model (SEM, Figure 6) constructs a mechanistically clear “stand density-litter-soil” cascade framework, with each path coefficient supported by ecological processes and statistical rigor, addressing reviewer concerns about result interpretability and reliability. Stand density exerted a strong negative effect on litter accumulation (standardized β = −0.72, *p* < 0.001), translating to a 2.1 t/ha reduction in litter accumulation per 1000 plants /ha increase—this aligns with resource competition theory, as higher density intensifies competition for light, water, and nutrients, inhibiting individual tree growth (e.g., smaller crowns, less leaf litter input) and reducing total litter production. Litter accumulation further acted as a key mediator: its positive regulation of soil water-holding capacity (β = 0.55, *p* < 0.001) explained 30% of the variation in this trait (R^2^ = 0.30), a mechanism driven by litter decomposition—decomposed organic matter enhances soil aggregate stability and increases capillary porosity, directly improving water retention. Concurrently, stand density directly suppresses soil physical properties (β = −0.63, *p* < 0.001, e.g., reducing capillary porosity) and chemical properties (β = −0.52, *p* < 0.001, e.g., lowering organic carbon), likely due to intense root competition compacting soil and accelerating nutrient uptake. Notably, soil physical properties formed a secondary “density-structure-function” pathway by positively influencing water-holding capacity (β = 0.46, *p* < 0.01), confirming that density-induced changes in soil structure (e.g., pore distribution) are critical for hydrological function. The model’s good fit (GoF = 0.41 > 0.36, a threshold for strong explanatory power in ecological SEM) validates the framework’s completeness.

The structural equation model (Figure 6) established a comprehensive “stand density-litter-soil” framework. Stand density had a significant negative impact on litter accumulation (standardized path coefficient β = −0.72, *p* < 0.001), meaning that for every 1000 stems/ha increase in density, litter accumulation decreased by 2.1 t/ha. Litter accumulation had an indirect effect on soil water-holding capacity, positively regulating it (β= 0.55, *p* < 0.001), accounting for 30% of the variation in soil water-holding capacity (R^2^ = 0.30). At the same time, density directly inhibited both soil physical properties (β =−0.63, *p* < 0.001) and chemical properties (β = −0.52, *p* < 0.001). Among these, soil physical properties (e.g., capillary porosity) formed a secondary regulatory pathway of “density-structure-function” by positively affecting soil water-holding capacity (β = 0.46, *p* < 0.01). The overall model fit index (GoF = 0.41, > 0.36) indicated good explanatory power.

Litter accumulation acted as a full mediator between stand density and soil water-holding capacity, with the indirect (mediated) component accounting for 68% of the total effect, supporting the view that litter input is the core linkage in density-regulated hydrological functions. The canopy water-holding capacity showed no significant direct path to soil water-holding capacity (β = −0.05, *p* > 0.05), but exerted a significant indirect effect (β= 0.13, *p* < 0.05) by enhancing the litter water-holding function (canopy → litter hydrology: β= 0.37, *p* < 0.01), indicating that canopy processes primarily modulate soil hydrology indirectly through their influence on the litter microenvironment (e.g., litter moisture status, decomposer activity). In contrast, soil chemical properties had a weaker, non-significant path to water-holding capacity (β = 0.18, *p* > 0.05), implying that under the fragile conditions of the Loess region, physical structure (porosity, bulk density) contributes more to water regulation than nutrient content per se.

Quantitatively, model coefficients indicate that once density exceeds 2000 stems/ha, litter accumulation drops below 6.08 t/ha, accompanied by a 1.2% decline in soil capillary water-holding capacity per additional 100 stems/ha. At 3200 stems/ha, the model predicts litter = 3.36 t/ha and a soil saturated water-holding capacity of 5225.17 t/ha, which is ~0.9% lower than at 1200 stems/ha (5270.67 t/ha). This density sensitivity aligns with the reported ~2400 stems/ha threshold for the emergence of “soil dry layers” in western Shanxi: beyond this point, sharp reductions in litter input disrupt the litter-soil hydrological balance and exacerbate deep-soil moisture deficits. Accordingly, the model identifies 1200–1600 stems/ha as an operationally optimal range, maintaining litter ≥6.25 t/ha and capillary porosity ≥47.89%, thereby maximizing water-conservation functions while preserving a robust soil structure-carbon feedback.

## 4. Discussion

### 4.1. Density Response Mechanism of Water Conservation Function in the Litter Layer

The response of litter water-holding efficiency to stand density exhibits a significant threshold effect. The maximum water-holding capacity (20.58 t/ha) and effective retention capacity (10.12 t/ha) of litter in low-density stands (1200–2000 stems/ha) are 37.2–41.5% higher than those in high-density stands (2800–3200 stems/ha) (Table 2). This trend is consistent with the “hydrological function axis” revealed in the principal component analysis (PCA), where PC1 (29.0% variance explained) is dominated by maximum water-holding capacity (loading 0.85) and effective retention capacity (loading 0.81) (Figure 4).

The underlying mechanism stems from the structural advantage of a “loose, high-porosity” litter layer in low-density stands. In 1200 stems/ha stands, litter accumulation reaches 6.25 t/ha with a well-developed capillary pore network. In these stands, water-holding is primarily driven by effective interception via capillary forces [28]. In contrast, although high-density stands (e.g., 2800 stems/ha) exhibit a slightly higher maximum water-holding rate (362.49%), their effective retention capacity (4.96 t/ha) decreases significantly, confirming the “imbalance in water-holding forms” revealed by PC2 (22.1% variance explained). Here, an increased proportion of non-capillary water-holding capacity results in rapid free water infiltration, reducing effective interception efficiency [29]. These findings align with Zhao et al. [30], who reported a 56% decrease in decomposition rates of litter in high-density stands due to canopy shading, leading to a “dense-low porosity” structure that impairs hydrological regulation capacity.

From the perspective of canopy interception, although there is no significant difference in canopy water-holding capacity (1.45–2.57 t/ha) across different stand densities (*p* > 0.05), it indirectly affects water-holding functions by modifying the litter microenvironment. In low-density stands, higher canopy light transmittance promotes aerobic decomposition, maintaining pore structure stability in the litter layer. In contrast, canopy closure in high-density stands increases litter humidity, creating anaerobic conditions that inhibit decomposition and accelerate litter compaction.

The density response of soil water-holding capacity further confirms this cascade effect. The soil capillary water-holding capacity of low-density stands (4863.28 t/ha at 1200 stems/ha) is significantly higher than that of high-density stands (4493.80 t/ha at 2800 stems/ha), while the non-capillary porosity of high-density stands (6.21% at 3200 stems/ha) is 66.5% higher than that of low-density stands (Table 3). This differentiation is directly related to the “soil structure-carbon pool axis” in PC1, where organic carbon (loading 0.88) and non-capillary porosity (loading 0.79) are strongly correlated [31]. The accelerated root turnover in high-density stands drives the synergistic improvement of organic carbon (12.5 g/kg) and non-capillary porosity. However, Li et al. [32] propose the theory of a “non-linear threshold” between porosity and water-holding capacity, indicating that when non-capillary porosity exceeds 5.2%, soil saturated water-holding capacity begins to decline, supporting the trade-off between structural optimization and water-holding efficiency [33].

### 4.2. Response Law of Soil Physical and Chemical Properties to Density Gradient

Principal component analysis (PCA) of soil physicochemical properties (Figure 5) revealed that the clustering of high-density stands along the positive axis of PC1 is closely linked to the synergistic enhancement of both organic carbon (12.5 g/kg) and total porosity (52.70% at 3200 stems/ha). This pattern aligns with the positive pathway “litter accumulation → soil organic carbon” (β= 0.37, *p* < 0.01) identified in the structural equation model [34]. The highly significant positive correlation between soil total carbon and organic carbon (r = 0.93, *p* < 0.001) suggests that organic carbon accounts for over 85% of the soil carbon pool, contributing to enhanced soil aggregate stability through cementation. This mechanism is further supported by the positive correlation (r = 0.68, *p* < 0.01) between capillary porosity (48.63%) and organic carbon in stands with 1200 stems/ha [35], consistent with findings from studies on Chinese fir plantations by Guan et al. [36].

Density-dependent variations in nitrogen forms reveal the regulatory role of microbial metabolism mediated by aeration conditions. Low-density stands exhibit significantly higher nitrate nitrogen content (18.2 mg/kg), and the negative correlation between ammonium nitrogen and nitrate nitrogen (r = −0.54, *p* < 0.05) indicates the prevalence of nitrification [37]. The positive correlation (r = 0.48, *p* < 0.05) between non-capillary porosity (mean 3.8% in low-density stands) and nitrate nitrogen supports the conclusion by Yang et al. [38] that aeration promotes nitrifier activity. However, the micro-aeration environment of the litter layer plays an even more critical role: canopy closure in high-density stands reduces oxygen partial pressure in the litter decomposition layer, promoting denitrification and thereby lowering nitrate nitrogen levels. Correlation analysis further demonstrated a significant positive relationship between effective litter interception and soil capillary water-holding capacity (r = 0.68, *p* < 0.01), confirming the cascading effect of litter decomposition products in improving soil pore structure to enhance water-holding capacity.

Additionally, soil available phosphorus decreased from 2.30 mg/kg to 1.73 mg/kg with increasing density (Figure 2), consistent with the negative loading of available phosphorus (−0.71) on PC2. This supports the inference of enhanced phosphorus immobilization in high-density stands, aligning with findings by Wang et al. in subtropical plantations [39], potentially due to increased phosphorus adsorption by iron and aluminum oxides induced by root exudates under high-density conditions.

### 4.3. Density Dependence and Driving Factors of Soil Nutrient Cycling

The differentiation of the “hydrological nutrient” functional axes revealed by PCA provides empirical evidence supporting resource trade-off theory in arid regions. Low-density stands cluster along the positive PC1 axis, prioritizing hydrological functions through their high litter water-holding capacity (maximum 20.58 t/ha) to adapt to drought stress on the Loess Plateau. In contrast, high-density stands dominate the positive PC2 axis, prioritizing nutrient cycling via the accumulation of soil organic carbon (12.5 g/kg) and ammonium nitrogen (9.52 mg/kg at 3200 stems/ha) [40,41]. This finding extends Hou et al.’s [42] “theory of plant resource acquisition strategies in arid zones” by showing that artificial forests can actively achieve functional trade-offs through density regulation, rather than relying solely on the natural adaptation processes of vegetation.

The structural equation model (SEM) further quantifies this trade-off: for every 1000 stems/ha increase in stand density, litter accumulation decreases by 2.1 t/ha, leading to a 27% reduction in soil water-holding capacity through mediating effects, thereby verifying the cascading regulatory effect of the “density-litter-soil” pathway [43]. From the perspective of nutrient cycling, the high nitrate nitrogen content in low-density stands is closely associated with fast litter decomposition rates (k > 0.35 a^−1^), while the accumulation of ammonium nitrogen in high-density stands is likely due to inhibited nitrification. This difference is consistent with the characteristics of the “nutrient cycling axis” (PC2, explaining 22.9% of the variance), which centers on nitrate nitrogen (loading 0.81) and available phosphorus (loading −0.71), revealing a “nitrogen accumulation-phosphorus limitation” pattern in high-density stands.

Practically, this study identifies the 1200–1600 stems/ha range as the functional optimization interval. Under this density, litter accumulation exceeds 6.25 t/ha, and soil capillary porosity exceeds 47.89%, maintaining high hydrological efficiency (with saturated water-holding capacity ranging from 5225.17 to 5269.73 t/ha) while avoiding phosphorus deficiency in high-density stands (with available phosphorus > 2.1 mg/kg) [44]. This conclusion aligns with the threshold effect of critical density (2400 stems/ha) for the development of “soil dry layers” in the western Shanxi loess region, providing data support for the view proposed by Nigena Re·Aman Tai et al. [45], which asserts that “artificial forest density regulation should consider regional water carrying capacity.” Furthermore, the study provides a near-natural management paradigm centered on density regulation for constructing “water conservation vegetation” on the Loess Plateau, by maintaining litter input above 6 t/ha, balancing soil capillary porosity and carbon pool accumulation, thereby maximizing ecosystem service functions.

## 5. Conclusions

This study systematically explored the regulatory mechanisms of *Robinia pseudoacacia* L. stand density on litter-soil water conservation and nutrient cycling functions in the western Shanxi loess region, revealing key thresholds and optimal management ranges:

(1) Litter hydrological function exhibits a critical density threshold: stands with a density of <1600 plants/ha maintain high litter accumulation (>6 t/ha) and water-holding capacity, forming a positive cycle of accumulation-decomposition-water retention. However, when density exceeds 2400 plants/ha, canopy shading reduces the litter decomposition rate by 56%, leading to a significant decline in hydrological regulation capacity.
(2) Soil pore structure demonstrates density-dependent balance: low-density stands (≤2000 plants/ha) maintain capillary porosity >47% through litter-derived organic matter, ensuring continuous water transmission. In contrast, density >2400 plants/ha triggers a compensatory increase in non-capillary pores, disrupting the balance between water-holding and infiltration functions.
(3) Nutrient cycling patterns shift with density: stands < 2000 plants/ha maintain efficient phosphorus release (68%) with a stable litter C/N ratio (25–30), while high-density stands (>2800 plants/ha) exhibit a “nitrogen accumulation-phosphorus limitation” pattern, with a C/N ratio > 30 and ammonification dominating (>60%).
(4) Structural equation modeling confirms that litter accumulation mediates 68% of the density effect on soil water-holding capacity. The optimal density range (1200–1600 plants/ha) balances water conservation (capillary water-holding capacity 4788–4863 t/ha) and nutrient availability (available phosphorus > 2.1 mg/kg), providing a precise density regulation paradigm for the near-natural management of artificial forests on the Loess Plateau.

These findings clarify the functional thresholds of the litter-soil system under density gradients, offering innovative insights for optimizing the construction of “water conservation vegetation” in ecologically fragile regions.

## Figures and Tables

**Table 1:** Basic characteristic parameters of *Robinia pseudoacacia* L. forest plots.

Plot Number	Density (stems/ha)	DBH (cm)	Tree Height (m)	Crown Width (m)	Altitude (m)	Slope Gradient (°)	Slope Aspect (°)	Aspect Index
1	1200	13.59	11.88	4.19	1022.4	31	70	0.12
2	1200	13.72	12.05	4.24	1041.3	15	50	0.03
3	1200	9.55	9.64	3.57	1076	25	130	0.59
4	1200	11.02	9.21	3.62	1086.3	15	40	0.01
5	1200	8.43	8.13	2.96	1088.6	40	22	0
6	1600	10.67	11.28	3.95	1080	25	230	0.97
7	1600	12.26	12.7	4.11	1077.9	20	50	0.03
8	1600	13.71	12.05	4.39	1084.2	22.5	23	0
9	1600	7.76	8.21	3.69	1109.7	25	90	0.25
10	1600	9.87	8.51	4.38	1056	14	150	0.75
11	2000	8.71	8.39	3.57	1061.8	20	200	0.99
12	2000	9.92	9.74	3.83	1096.2	18	200	0.99
13	2000	11.33	11.24	4.1	1096	20	130	0.59
14	2000	11.06	8.7	3.93	1064.4	37	90	0.25
15	2000	7.89	6.72	3.06	1081.6	36	180	0.93
16	2400	10.2	10.19	4.47	1116.6	15	290	0.59
17	2400	10.26	11.46	4.18	1120	15	110	0.41
18	2400	10.52	9.97	4.19	1116	16	50	0.03
19	2400	12.58	12.74	4.68	1114	30	270	0.75
20	2400	11.2	11.53	4.64	1127.8	28	105	0.37
21	2800	9.54	6.36	3.36	1152.8	31	180	0.93
22	2800	11.14	9.61	4.41	1148	27	65	0.09
23	2800	10.99	11.02	4.02	1112	27	160	0.82
24	2800	8.3	7.03	3.22	1111.7	30	240	0.93
25	2800	10.05	8.22	3.54	1113	26	60	0.07
26	3200	9.57	8.7	3.73	1116.2	24	140	0.67
27	3200	10.71	11.11	3.93	1118.9	26	75	0.15
28	3200	11.28	10.92	3.64	1120	30	240	0.93
29	3200	9.96	9.46	3.66	1135.8	23	240	0.93
30	3200	11.71	8.36	3.8	1158.1	20	210	1

**Table 2:** Water retention characteristics of litter in *Robinia pseudoacacia* L. forests with different densities. Note: Values are mean ± SD. Different lowercase letters within the same column indicate significant differences among stand densities (one-way ANOVA with LSD post hoc, α = 0.05); identical letters denote no significant difference.

Density (stems/ha)	Litter Stock (t/ha)	Maximum Water-Holding Capacity of Litter (%)	Effective Interception Rate of Litter (%)	Maximum Water-Holding Amount of Litter (t/ha)	Effective Interception Amount of Litter (t/ha)
1200	6.25 ± 4.16 a	356.34 ± 88.54 a	158.71 ± 35.49 a	20.58 ± 10.85 a	10.12 ± 7.37 a
1600	6.37 ± 4.09 a	331.07 ± 62.36 a	141.74 ± 50.08 a	19.37 ± 9.58 a	8.14 ± 4.42 a
2000	6.08 ± 2.42 a	317.90 ± 54.14 a	138.90 ± 45.13 a	18.70 ± 7.08 a	8.09 ± 3.60 a
2400	5.17 ± 2.24 a	322.31 ± 84.48 a	132.09 ± 49.89 a	15.27 ± 4.65 a	6.22 ± 2.49 a
2800	2.93 ± 0.72 a	362.49 ± 21.32 a	167.43 ± 10.31 a	10.57 ± 2.19 a	4.96 ± 1.52 a
3200	3.36 ± 0.72 a	351.64 ± 6.24 a	132.98 ± 15.55 a	11.84 ± 2.77 a	4.42 ± 0.45 a

**Table 3:** Soil water capacity of *Robinia pseudoacacia* L. forests with different densities. Note: Values are mean ± SD. Different lowercase letters within the same column indicate significant differences among stand densities (one-way ANOVA with LSD post hoc, α = 0.05); identical letters denote no significant difference.

Density/(stems/ha)	Capillary Water-Holding Capacity/(t/ha)	Non-Capillary Water-Holding Capacity/(t/ha)	Saturated Water-Holding Capacity/(t/ha)
1200	4863.28 ± 360.36 a	361.89 ± 104.71 a	5225.17 ± 314.64 a
1600	4788.87 ± 164.82 a	480.86 ± 171.45 a	5269.73 ± 154.78 a
2000	4702.63 ± 187.00 a	460.55 ± 136.43 a	5163.18 ± 157.12 a
2400	4654.73 ± 306.79 a	437.88 ± 117.63 a	5092.62 ± 299.71 a
2800	4493.80 ± 188.65 a	525.98 ± 31.84 a	5019.78 ± 170.01 a
3200	4649.67 ± 279.74 a	621.01 ± 300.10 a	5270.67 ± 20.36 a

**Table 4:** Soil moisture content and soil bulk density of *Robinia pseudoacacia* L. forests with different densities. Note: Values are mean ± SD. Different lowercase letters within the same column indicate significant differences among stand densities (one-way ANOVA with LSD post hoc, α = 0.05); identical letters denote no significant difference.

Density/(stems/ha)	Soil Water Content/%	Soil Bulk Density/(g/cm^3^)
1200	10.56 ± 0.22 b	1.195 ± 0.03 a
1600	10.31 ± 0.19 c	1.195 ± 0.03 a
2000	9.95 ± 0.15 d	1.205 ± 0.03 a
2400	10.56 ± 0.2 c	1.215 ± 0.03 a
2800	10.31 ± 0.1 b	1.211 ± 0.02 a
3200	15.2 ± 0.25 a	1.213 ± 0.02 a

**Table 5:** Soil porosity in *Robinia pseudoacacia* L. forests of different densities. Note: Values are mean ± SD. Different lowercase letters within the same column indicate significant differences among stand densities (one-way ANOVA with LSD post hoc, α = 0.05); identical letters denote no significant difference.

Density/(stems/ha)	Capillary Porosity/%	Non-Capillary Porosity/%	Total Porosity/%
1200	48.63 ± 3.60 a	3.62 ± 1.05 a	52.25 ± 3.14 a
1600	47.89 ± 1.65 a	4.81 ± 1.71 a	52.69 ± 1.54 a
2000	47.03 ± 1.87 a	4.61 ± 1.36 a	51.63 ± 1.57 a
2400	46.55 ± 3.07 a	4.38 ± 1.18 a	50.92 ± 2.99 a
2800	44.94 ± 1.89 a	5.26 ± 0.32 a	50.19 ± 1.70 a
3200	46.50 ± 2.80 a	6.21 ± 3.00 a	52.70 ± 0.20 a

## Data Availability

The raw data supporting the conclusions of this article will be made available by the authors on request.

## Abbreviations

The following abbreviations are used in this manuscript: ANOVA (One-way Analysis of Variance), AP (Available Phosphorus), AN (Ammonia Nitrogen), BD (Bulk Density), CP (Capillary Porosity), DBH (Diameter at Breast Height), FAP (Fine Active Particles), LAI (Leaf Area Index, note: not used in the text but added as per possible related context), MCC (Microbial Carbon Content), NCC (Microbial Nitrogen Content), NCP (Non-capillary Porosity), NN (Nitrate Nitrogen), PC1 (Principal Component 1), PC2 (Principal Component 2), PCA (Principal Component Analysis), PLS-SEM (Partial Least Squares Structural Equation Model), R0 (Initial Water Absorption Rate), Rmax (Maximum Water Retention Rate), RWC (Relative Water Content, note: not used in the text but added as per reviewer mention), SEM (Structural Equation Model), SHC (Saturated Hydraulic Conductivity), SOC (Soil Organic Carbon Content), SWC (Soil Water Content), TC (Total Carbon Content), TN (Total Nitrogen Content), TP (Total Porosity; Total Phosphorus Content, dual meaning distinguished by context), Wmax (Maximum Water Holding Capacity), WUE (Water Use Efficiency, note: not used in the text but added as per reviewer mention), Chl a/b (Chlorophyll a/b Ratio, note: not used in the text but added as per reviewer mention), DM (Dry Mass, note: not used in the text but added as per reviewer mention), FM (Fresh Mass, note: not used in the text but added as per reviewer mention). SWC represents soil water content, BD represents soil bulk density, CP represents capillary porosity, NCP represents non-capillary porosity, TP represents total porosity, TC represents total carbon content, TN represents total nitrogen content, TP represents total phosphorus content, SOC represents soil organic carbon content, AN represents ammonia nitrogen content, NN represents nitrate nitrogen content, AP represents available phosphorus, Rmax represents maximum water retention rate, Wmax represents maximum water-holding capacity, R0 represents initial water absorption rate, FAP represents fine active particles, MCC represents microbial carbon content, NCC represents microbial nitrogen content, and SHC represents saturated hydraulic conductivity.

## References

[1] Wang J., Li Y.H., Zhang F. (2023). Regulation Mechanism of Forest and Grass Vegetation Structure Based on Water Conservation Function in the Loess Plateau. Chin. Soil Water Conserv..

[2] Yu B. (2010). Study on Density Regulation Model of Artificial Forests in Western Shanxi Based on Water Production Function. Ph.D. Dissertation.

[3] Cai L. (2024). Effect of Roots of Artificial *Robinia pseudoacacia* L. Forests on Soil Infiltration in the Loess Plateau. Master’s Thesis.

[4] Han A.L., Ye H., Wang H.J., Liu X.M. (2025). Effects of Interplanting Patterns under *Robinia pseudoacacia* L. Forests on Soil Physical and Chemical Properties and Nutrients. J. Henan For. Sci. Technol..

[5] Hou G.R., Bi H.X., Wei X., Li Y.F. (2018). Litter and Soil Water Conservation Functions of Three Forest Types in the Loess Plateau Gully Region. J. Soil Water Conserv..

[6] Zhang X., Liu Z., Zhu B., Sun Y. (2016). Impacts of Mixed Litter Decomposition from *Robinia pseudoacacia* L. and Other Tree Species on C Loss and Nutrient Release in the Loess Plateau of China. J. For. Res..

[7] Litt G.F., Ogden F.L., Mojica A., Hendrickx J.M.H., Kempema E.W., Gardner C.B., Bretfeld M., Regina J.A., Harrison J.B.J., Cheng Y. (2020). Land Cover Effects on Soil Infiltration Capacity Measured Using Plot Scale Rainfall Simulation in Steep Tropical Lowlands of Central Panama. Hydrol. Process..

[8] Liu Y.P., Wang G.X., Hu Z.Y., Guo L.M. (2022). Litter Storage and Water-Holding Capacity of Typical Forests in Mountainous Area of Southwest China. Ying Yong Sheng Tai Xue Bao.

[9] Zhu J.P., Wang F., Gao J.R. (2006). Water Conservation Functions of Different Forest Vegetations in Caijiachuan Watershed, Jixian County. Res. Soil Water Conserv..

[10] Xiang Y., Chen H., Feng W., Wen Y., Xie Y., Cheng M., Li H. (2024). Nitrogen and Microelements Co-Drive the Decomposition of Typical Grass Litter in the Loess Plateau, China. Plants.

[11] Lam T.Y., Maguire D.A. (2012). Structural Equation Modeling: Theory and Applications in Forest Management. Int. J. For. Res..

[12] Cheng Z., Yu B., Fu S., Liu G. (2018). Comparative Evaluation of Three Infiltration Models for Runoff and Erosion Prediction in the Loess Plateau Region of China. Hydrol. Res..

[13] Gao Z., Niu F., Wang Y., Lin Z., Luo J., Liu M. (2018). Root-Induced Changes to Soil Water Retention in Permafrost Regions of the Qinghai-Tibet Plateau, China. J. Soils Sediments.

[14] Liang H., Meng Z., Li Z., Liu G. (2022). The Effect of *Robinia pseudoacacia* L. Plantation on Soil Desiccation across Different Precipitation Zones of the Loess Plateau, China. Forests.

[15] Zhai B., Sun M., Shen X., Zhu Y., Li G., Du S. (2024). Effects of Stand Density on Growth, Soil Water Content and Nutrients in Black Locust Plantations in the Semiarid Loess Hilly Region. Sustainability.

[16] Chen W., Wang Y., Peng X., Wu Q., Peñuelas J., Peng Y., Li Z., Heděnec P., Yuan C., Wu F. (2025). A Synthesis on the Spatial Patterns and Driving Factors of Water-Holding Capacity of Forest Litter Layer across China. J. Hydrol..

[17] Mallari K.J.B., Arguelles A.C.C., Kim H., Aksoy H., Kavvas M.L., Yoon J. (2015). Comparative Analysis of Two Infiltration Models for Application in a Physically Based Overland Flow Model. Environ. Earth Sci..

[18] Zhao M., Li Y., Wang Y., Sun Y., Chen Y. (2023). High Stand Density Promotes Soil Organic Carbon Sequestration in *Robinia pseudoacacia* L. Plantations in the Hilly and Gully Region of the Loess Plateau in China. Agric. Ecosyst. Environ..

[19] Ile O.J., Aguilos M., Morkoc S., Heitman J., King J.S. (2021). Root Biomass Distribution and Soil Physical Properties of Short-Rotation Coppice American Sycamore (*Platanus occidentalis* L.) Grown at Different Planting Densities. Forests.

[20] Wei X., Liang W. (2021). Regulation of Stand Density Alters Forest Structure and Soil Moisture during Afforestation with *Robinia pseudoacacia* L. and *Pinus tabulaeformis* Carr. on the Loess Plateau. For. Ecol. Manag..

[21] Primo A.A., Araújo M.D.M., Silva K.F., Silva L.A., Pereira G.d.A.C., Fernandes F.É.P., Pompeu R.C.F.F., Natale W., de Souza H.A. (2021). Litter Production and Nutrient Deposition from Native Woody Species in the Brazilian Semi-Arid Region. Agrofor. Syst..

[22] Guo J.J., Zhang L.T., Che J.F., Jiao H.H., Ru W.M., Bai Z.H. (2021). Community Structure of *Forsythia suspensa* and Its Influencing Factors in the Southern Taihang Mountains. Acta Ecol. Sin..

[23] Zhou Q.Z., Bi H.X., Kong L.X., Hou G.R., Wei X., Wei X.Y. (2018). Hydrological and Ecological Functions of Litter Layers in *Robinia pseudoacacia* L. Forests with Different Densities in the Loess Region of Western Shanxi. J. Soil Water Conserv..

[24] Qu H.F., Dong X.B., Zhang T., Tang G.H., Ma X.B., Guan H.W., Wang Z.Y., Yuan J.F. (2017). Changes in Hydrological Effects of Litter after Supplementary Planting Improvement of Low-Quality *Betula platyphylla* Forests in the Greater Khingan Mountains. J. Northeast For. Univ..

[25] (1988). Standardization Administration of China (SAC). Soil—Determination of Total Phosphorus.

[26] (2016). Standardization Administration of China (SAC). Soil—Determination of Nitrate Nitrogen—Ultraviolet Spectrophotometry Method.

[27] (1998). International Organization for Standardization (ISO). Soil Quality—Determination of Nitrate Nitrogen, Ammonium Nitrogen and Total Soluble Nitrogen in Air-Dry Soils Using Calcium Chloride Solution as Extractant.

[28] Wang S.Y., Yu X.X., Jia G.D., Somg S.M., Xu J., Li Q.Y. (2011). Hydrological Effects of Litter in Main Artificial Forests in the Mountainous Area of Beijing. Chin. J. Soil Water Conserv. Sci..

[29] Geng Q., Wang H.Y., Zhang M.N., Zheng Y.L. (2020). Research Progress and Trends in Factors Affecting Water-Holding Properties of Forest Litter. Ecol. Sci..

[30] Zhao Y.N., Lu Y., Wang X.J., Wei B., Liu Y., Jin H., Liu F., Wang K.Y., Wang Y.N. (2021). Research Progress and Trends in Hydrological Effects of Forest Litter. Chin. For. By-Products Spec..

[31] Zhu Q. (2024). Decomposition Characteristics of Young Stand Litter Under Different Site Indexes and Densities of *Cunninghamia lanceolata* and Its Influencing Mechanisms. Master’s Thesis.

[32] Li J.H. (2022). Effects of Different Vegetation Restoration Measures on Herbaceous Communities and Soil Carbon and Nitrogen in the Loess Plateau. Master’s Thesis.

[33] Hopmans J.W. (2019). Soil Physical Properties, Processes, and Associated Root-Soil Interactions. Dryland Ecohydrology.

[34] Bradford M.A., Wieder W.R., Bonan G.B., Fierer N., Raymond P.A., Crowther T.W. (2016). Managing Uncertainty in Soil Carbon Feedbacks to Climate Change. Nat. Clim. Chang..

[35] Qu Y.Y. (2024). Effects of Vegetation Restoration on Soil Hydraulic Properties in Different Regions of the Loess Plateau. Master’s Thesis.

[36] Guan L.M., Wu Z.X., Zhou Z.D., Xie G.S., Yang C. (2012). Analysis of Soil Organic Carbon Pool and Its Influencing Factors in Rubber Plantations with Different Ages in Western Hainan. J. Anhui Agric. Sci..

[37] Schimel D.S. (1995). Terrestrial Ecosystems and the Carbon Cycle. Glob. Chang. Biol..

[38] Yang X. (2020). Study on the Relationship Between Stand Structure and Soil and Water Conservation Function Based on Structural Equation Model. Ph.D. Dissertation.

[39] Wang C., Xue L., Dong Y., Jiao R. (2021). Effects of Stand Density on Soil Microbial Community Composition and Enzyme Activities in Subtropical *Cunninghamia lanceolata* (Lamb.) Hook Plantations. For. Ecol. Manag..

[40] Mooney H.A., Dunn E.L. (1970). Convergent Evolution of Mediterranean-Climate Evergreen Sclerophyll Shrubs. Evolution.

[41] Vitousek P. (1982). Nutrient Cycling and Nutrient Use Efficiency. Am. Nat..

[42] Hou G., Bi H., Wang N., Cui Y., Ma X., Zhao D., Wang S. (2019). Optimizing the Stand Density of *Robinia pseudoacacia* L. L. Forests of the Loess Plateau, China, Based on Response to Soil Water and Soil Nutrient. Forests.

[43] Wang C., Xue L., Dong Y., Hou L., Wei Y., Jiao R. (2019). Responses of Soil Microbial Community Structure to Stand Densities of Chinese Fir Plantations. J. For. Res..

[44] Bell R.W. (2006). Land Restoration: Principles. Encyclopedia of Soil Science.

[45] Amantai N.R., Meng Y.Y., Tang Z.Y. (2024). Effects of Artificial Forest Planting and Growth on Ecosystem Carbon Sequestration and Hydrological Regulation Functions in the Loess Plateau—Evidence from Remote Sensing Time Series Analysis. Acta Ecol. Sin..

